# Long noncoding RNAs as potential diagnostic biomarkers for diabetes mellitus and complications: A systematic review and meta‐analysis

**DOI:** 10.1111/1753-0407.13510

**Published:** 2023-12-23

**Authors:** Xuee Su, Huibin Huang, Jinqing Lai, Shu Lin, Yinqiong Huang

**Affiliations:** ^1^ Centre of Neurological and Metabolic Research The Second Affiliated Hospital of Fujian Medical University Quanzhou China; ^2^ Department of Anaesthesia The Second Affiliated Hospital of Fujian Medical University Quanzhou China; ^3^ Department of Endocrinology The Second Affiliated Hospital of Fujian Medical University Quanzhou China; ^4^ Department of Neurosurgery The Second Affiliated Hospital of Fujian Medical University Quanzhou China; ^5^ Obesity and Metabolic Disease Research Group Garvan Institute of Medical Research Sydney New South Wales Australia

**Keywords:** diabetic nephropathy, diabetic retinopathy, long noncoding RNA, meta‐analysis, type 2 diabetes mellitus

## Abstract

**Aims:**

Long noncoding RNAs (lncRNAs) may be associated with the development of type 2 diabetes mellitus and its complications; however, the findings remain controversial. We aimed to synthesize the available data to assess the diagnostic utility of lncRNAs for identification of type 2 diabetes mellitus and its consequences.

**Materials and Methods:**

We performed a systematic review and meta‐analysis, searching PubMed, Embase, and Web of Science for articles published from September 11, 2015 to December 27, 2022. We evaluated human case–control or cohort studies on differential lncRNA expression in type 2 diabetes mellitus or its associated comorbidities. We excluded studies if they were non‐peer reviewed or published in languages other than English. From 2387 identified studies, we included 17 (4685 participants).

**Results:**

Analysis of the pooled data showed that lncRNAs had a diagnostic area under the curve (AUC) of 0.84 (95% CI: 0.80–0.87), with a sensitivity of 0.79 (95% CI: 0.74–0.83) and a specificity of 0.75 (95% CI: 0.69–0.80). LncRNAs had an AUC of 0.65 for the diagnosis of prediabetes, with 82% sensitivity and 65% specificity.

**Conclusions:**

LncRNAs may be promising diagnostic markers for type 2 diabetes mellitus and its complications.

## Graphical abstract

**Highlights**

- Long noncoding RNAs (lncRNAs) may be associated with type 2 diabetes mellitus (T2DM) and related complications.
- We performed a systematic review and meta‐analysis of related published literature.
- LncRNAs had T2DM diagnostic area under the curve of 0.84, sensitivity of 0.79, and specificity of 0.75.
- These values for diagnosing prediabetes were 0.65%, 82%, and 65%, respectively.
- Thus, lncRNAs may be promising diagnostic markers for T2DM and related complications.

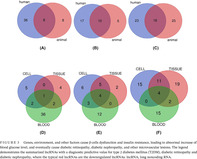

## 1 INTRODUCTION

Diabetes mellitus (DM) is a global health crisis and is associated with increased risk of heart disease and stroke; 90%–95% are type 2 diabetes mellitus (T2DM), which is characterized by increasing beta‐cell loss and insulin resistance, predominantly caused by obesity.^1^ By 2045, 700 million individuals are expected to develop diabetes, with 463 million currently living with the disease worldwide.^2^ The disease is caused by multiple factors, including genetics, immune responses, oxidative stress, and endoplasmic reticulum stress.^3^ Persistently elevated blood glucose levels damage organs and tissues, leading to the development of diabetic complications, such as microvascular disease, diabetic retinopathy (DR), and diabetic nephropathy (DN).^4^, ^5^ DR is the most common manifestation of diabetic microangiopathy worldwide. Approximately 191 million people are expected to have DR by 2030, which potentially results in blindness and has a significant impact on the quality of life of those who are affected.^6^ DN is another common microvascular complication of diabetes, affecting approximately 3% of the population.^7^ The diagnosis and treatment of T2DM and its complications have made tremendous strides in recent years owing to the rapid improvement of healthcare systems; yet, the treatment outcomes for this chronic disease remain unsatisfactory. Thus, identifying preventive measures and new biomarkers for the early detection of disease through noninvasive tests is essential to prevent deterioration of beta‐cell function and preserve remaining beta‐cell mass.

Non‐protein‐coding RNA molecules with a length longer than 200 nucleotides are known as long noncoding RNAs (lncRNAs).^8^ With the rapid development in microarray technologies and high‐throughput sequencing, the biological properties and functions of lncRNAs are gradually being recognized. The regulation of various cellular responses and diseases is significantly affected by lncRNAs. Nowadays, noncoding RNAs have emerged as a diagnostic and prognostic biomarker for various diseases, such as cancer, infectious diseases, and autoimmune disorders.^9^, ^10^, ^11^ According to early studies, diabetes development is linked to lncRNAs with distinct expression patterns that also play a role in other endocrine functions and diseases.^12^ For example, Wang et al^13^ found that a characteristic feature of T2DM is increased expression of HOX antisense intergenic RNA, a type of lncRNA. High serum HOX antisense intergenic RNA expression is a promising noninvasive diagnostic marker and an independent predictor of T2DM. Moreover, many lncRNAs are aberrantly expressed with T2DM complications, such as DN and DR, and are also closely related to their pathogenesis.^14^, ^15^

Because of their differential regulation, methylation, and other mechanisms,^16^ lncRNAs are anticipated to contribute to the early detection and treatment of T2DM and are attracting research attention. Thus, we aimed to assess lncRNAs as potential biomarkers for T2DM and associated comorbidities by conducting a systematic review and meta‐analysis.

## 2 METHODS

### 2.1 Study design

This systematic review and meta‐analysis was part of a thematic project conducted at the Second Affiliated Hospital of Fujian Medical University. The study protocol was registered in the International Prospective Register of Systematic Reviews database (registration number, 42022381403), following the Preferred Reporting Items for Systematic Reviews and Meta‐Analyses 2020 guidelines for the reporting of systematic reviews.^17^

### 2.2 Search strategy and selection criteria

We searched PubMed, Embase, and Web of Science from their inception until December 27, 2022. The reference lists of key reviews and meta‐analyses were searched to supplement the identified citations. Our search strategy is available in Supplementary S3.

Eligible studies met the following inclusion criteria: (a) case–control or cohort studies on differential lncRNA expression in patients with T2DM or related complications; (b) included diabetic and nondiabetic patients, diabetes‐related complications, and compared diabetic and nondiabetic control samples; (3) contained data on the total number of samples, area under the receiver operating characteristic (ROC) curve (AUC), sensitivity, and specificity. We excluded studies unrelated to lncRNAs or T2DM, review papers, animal studies, case reports, those based on nonhuman subjects, or studies published in languages other than English.

### 2.3 Data analysis

#### 2.3.1 Study selection

After removing duplicates using EndNote X9 (Clarivate Analytics, Philadelphia, PA, USA), two team members (XES and JQL) further scanned the titles and abstracts independently, read the relevant full‐text manuscripts, and extracted the study data from eligible studies. Any disagreements were settled through conversation and, if necessary, through adjudication by a third team member (YQH). Furthermore, the reasons for exclusion were recorded. Data from the screening process were fully documented, including author, country, year of publication, lncRNA type, disease type, participants, method of detection, lncRNA expression levels, diagnostic power, sensitivity, and specificity.

#### 2.3.2 Data extraction and management

After consensus was reached, the data were entered into STATA statistical software 16.0 (Stata Corp., College Station, Texas, USA). We included the sample size, AUC, sensitivity, and specificity values from the original literature and calculated the following values: true positive, false positive, false negative, and true negative. An independent evaluation of the literature quality was conducted using the Newcastle‐Ottawa Scale,^18^ and any uncertainties were discussed and highlighted. Each article received a score between 0 and 9, with scores of 6 and 9 denoting exceptional quality.

Data were transferred to STATA statistical software 16.0 for meta‐analysis using the random‐effects mode. Heterogeneity between trials was assessed using the *I* ^2^ statistic, which describes between‐study variation as a percentage of the total variation. If the *I* ^*2*^ score was <50%, heterogeneity between the eligible studies was considered insignificant.^19^ For the pooled analysis, a fixed‐effects model was employed; however, when heterogeneity was considerable, a random‐effects model was applied. This was determined using a subgroup analysis of potential sources of survey heterogeneity. Deeks' funnel plot was the primary tool for assessment of publication bias, and a two‐tailed *p* value of .05 was considered significant. The outcomes were expressed as the AUC, which can be used to determine the decision process for the best model and allows for a quantitative assessment of the accuracy and practical utility of marker classification prediction. In general, when 0.5 < AUC <1, the classifier used outperforms the random prediction, as the random prediction provides a good predictive value when a good threshold is set, indicating that the point on the ROC curve is remarkably close to the (0,1) point. An AUC of 0.5 signifies no distinction; 0.7–0.8 is regarded as fair; 0.8–0.9 is viewed as great; and 0.9 is considered remarkable.^20^

#### 2.3.3 Measurement of the effects

The outcomes are presented as AUCs. The AUC values corresponding to lncRNA expression levels in diabetes and diabetes‐related complications were calculated.

#### 2.3.4 Missing data

Missing data were obtained by contacting relevant study authors. Studies with missing data were included when the specific data requested were sufficient for meta‐analysis; otherwise, only the reported results were considered.

#### 2.3.5 Subgroup analysis

We performed analyses to reduce heterogeneity and assess the effect of lncRNAs in patients with diabetes or its complications according to the following subgroups: (a) disease type (diabetes or prediabetes); (b) sample source, including serum, plasma, whole blood, and peripheral blood mononuclear cells (PBMCs); (c) lncRNA expression level (upregulated or downregulated); and (d) ethnicity of the included population.

#### 2.3.6 Sensitivity analysis

To determine the reliability of the study findings, a sensitivity analysis of the sample size was conducted. The stability of the results was assessed using repeated meta‐analyses to exclude publications that had already been included.

## 3 RESULTS

### 3.1 Literature search and study characteristics

**FIGURE 1:**
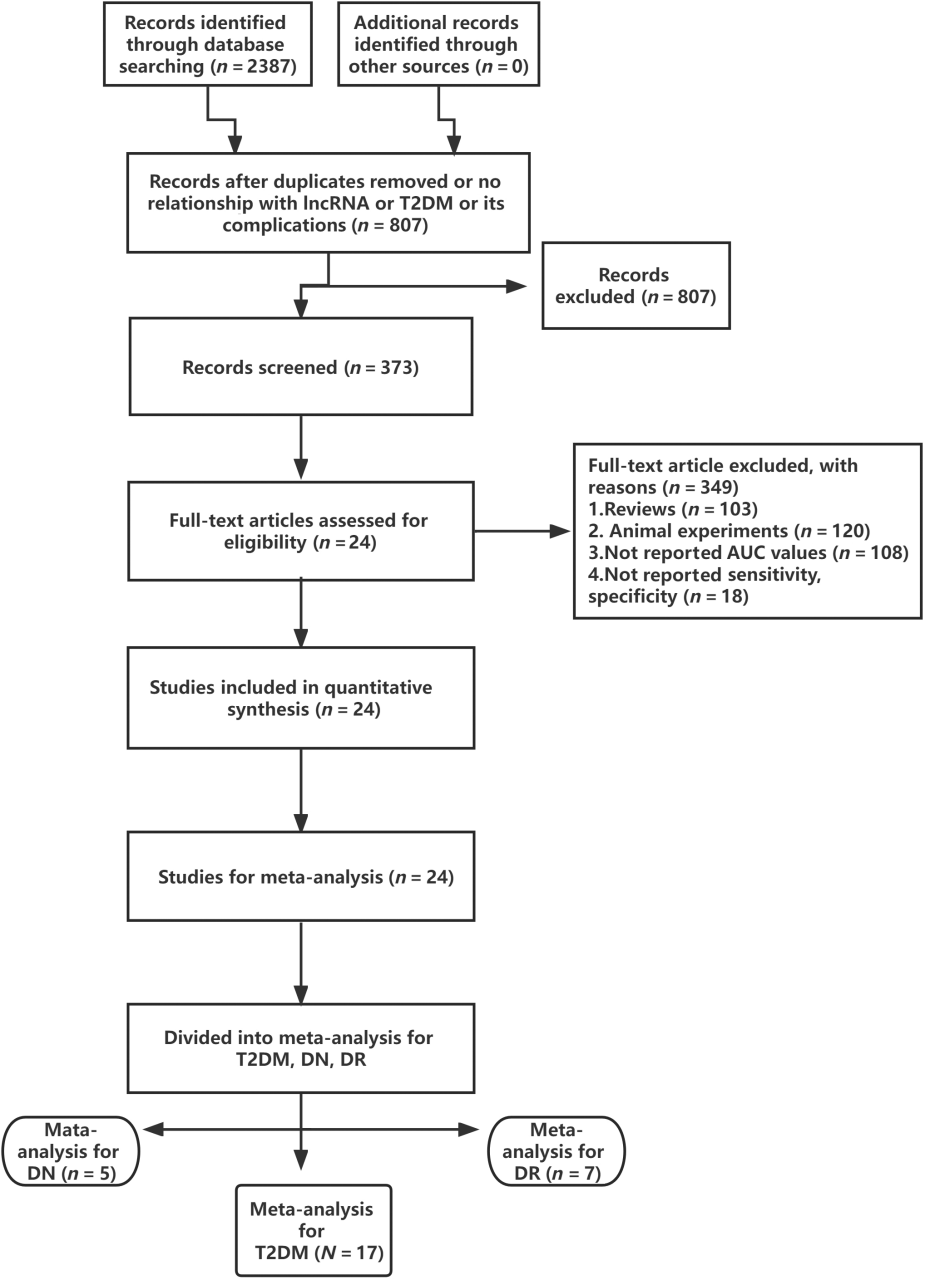
Flow chart of this study. AUC, area under the curve; DN, diabetic nephropathy; DR, diabetic retinopathy; lncRNA, long noncoding RNA; T2DM, type 2 diabetes mellitus.

The methodology for selecting the studies is shown in Figure 1. A total of 2387 potentially relevant studies were identified in our online database, 807 of which were excluded owing to duplicate titles and abstract assessments or irrelevance to lncRNA, T2DM, or its complications. The remaining 373 articles were thoroughly evaluated. In addition to the 103 review articles, a total of 246 studies were removed for the following reasons: 120 were animal studies, 108 did not report AUC values, and 18 did not report numerical sensitivity or specificity results. In total, 24 studies ultimately met our eligibility criteria, of which T2DM, DN, and DR were analyzed in 17,^21^, ^22^, ^23^, ^24^, ^25^, ^26^, ^27^, ^28^, ^29^, ^30^, ^31^, ^32^, ^33^, ^34^ 5,^5^, ^30^, ^35^, ^36^, ^37^ and 7 studies,^4^, ^28^, ^29^, ^32^, ^37^, ^38^, ^39^ respectively (some articles included more than one of these outcomes). Table 1 provides the specifics of the survey characteristics and summarizes the quality assessment results. The detail scores of Newcastle‐Ottawa Scale for quality of each included article are shown in Supplementary S4.

**TABLE 1:** Main characteristics of the included studies.

Study (ref)	Year	Country	LncRNA	Disease type	Sample size		Specimen	Method	Regulation	Diagnostic power			NOS score
					Case	Control				Sen	Spe	AUC	
Omidvar et al	2018	Iran	lncRNA VIM‐AS1	T2DM	100	100	PBMCs	qRT‐PCR	Down	0.56	0.68	0.63	7
Omidvar et al	2018	Iran	lncRNA CTBP1‐AS2	T2DM	100	100	PBMCs	qRT‐PCR	Down	0.59	0.75	0.68	7
Saeidi et al	2018	Iran	lncRNA LY86‐AS1	T2DM	100	98	PBMCs	qRT‐PCR	Down	0.65	0.80	0.747	7
Saeidi et al	2018	Iran	lncRNA HCG27_201	T2DM	100	98	PBMCs	qRT‐PCR	Down	0.56	0.75	0.65	7
Mansoori et al	2018	Iran	lncRNA LINC00523	T2DM	100	98	PBMCs	qRT‐PCR	Down	0.81	0.61	0.74	7
Mansoori et al	2018	Iran	lncRNA LINC00994	T2DM	100	98	PBMCs	qRT‐PCR	Down	0.81	0.53	0.67	7
Wang et al	2018	China	lncRNA CASC2	T2DM	296	56	Blood	qRT‐PCR	Down	0.66	0.48	0.63	6
Yin et al	2017	China	lncRNA GAS5	T2DM	10	30	Plasma	qRT‐PCR	Down	0.7	0.6	0.74	7
Carter et al	2015	United States	lncRNA GAS5	T2DM	47	49	Blood	qRT‐PCR	Down	0.85	0.67	0.81	7
Li et al	2017	China	lncRNA ENST00000550337.1	T2DM	64	60	Blood	qRT‐PCR	Up	0.75	0.65	0.73	6
Li et al	2017	China	lncRNA ENST 00000550337.1	Prediabetes	63	60	Blood	qRT‐PCR	Up	0.75	0.65	0.71	6
Li et al	2017	China	lncRNA TCONS_00007244	T2DM	20	20	Blood	qRT‐PCR	Up	0.75	0.65	0.71	6
Li et al	2017	China	lncRNA TCONS_00007244	Prediabetes	20	20	Blood	qRT‐PCR	Up	0.65	0.75	0.69	6
Li et al	2017	China	lncRNA TCONS_00000886	T2DM	20	20	Blood	qRT‐PCR	Up	0.85	0.6	0.7	6
Li et al	2017	China	lncRNA TCONS_00000886	Prediabetes	20	20	Blood	qRT‐PCR	Up	0.9	0.5	0.69	6
Shaker et al	2018	Egypt	LncRNA HOTAIR	T2DM	30	81	Serum	qRT‐PCR	Up	0.93	0.65	0.72	7
Shaker et al	2018	Egypt	LncRNA MALAT1	T2DM	30	81	Serum	qRT‐PCR	Up	0.57	0.69	0.64	7
Toraih et al	2019	Egypt	lncRNA CDKN2B‐AS1	T2DM	55	108	Plasma	qRT‐PCR	Up	0.70	0.60	0.73	6
Toraih et al	2019	Egypt	lncRNA PVT1	T2DM	55	108	Plasma	qRT‐PCR	Up	0.83	0.60	0.74	6
Cai et al	2021	China	lncRNA SNHG5	T2DM	62	60	Serum	qRT‐PCR	Up	0.81	0.83	0.90	7
Cao et al	2020	China	lncRNA LINC‐P21	T2DM	108	68	Serum	qRT‐PCR	Up	0.83	0.94	0.92	6
Ji et al	2022	China	lncRNA NR2F1‐AS1	T2DM	111	106	Blood	qRT‐PCR	Up	0.7664	0.6667	0.789	7
Su et al	2022	China	lncRNA TUG1	T2DM	105	105	Serum	qRT‐PCR	Up	0.63	0.97	0.64	7
Su et al	2022	China	lncRNA MEG3	T2DM	105	105	Serum	qRT‐PCR	Up	0.58	0.94	0.59	7
Ma et al	2021	China	ENST00000588707.1	T2DM	127	130	PBMCs	qRT‐PCR	Down	0.72	0.80	0.82	7
Ma et al	2021	China	TCONS_00004187	T2DM	127	130	PBMCs	qRT‐PCR	Down	0.82	0.61	0.83	7
Wang et al	2021	China	lncRNA HOTAIR	T2DM	96	82	Serum	qRT‐PCR	Up	0.87	0.84	0.90	8
Saleh et al	2020	Egypt	lncRNA HCG27_201	T2DM	70	70	Plasma	qRT‐PCR	Down	0.96	0.83	0.96	7
Saleh et al	2020	Egypt	lncRNA GAS5	T2DM	70	70	Plasma	qRT‐PCR	Down	0.96	0.76	0.94	7
Saleh et al	2020	Egypt	lncRNA LY86‐AS1	T2DM	70	70	Plasma	qRT‐PCR	Down	0.93	0.69	0.83	7
Saleh et al	2020	Egypt	lncRNA HCG27_201	Prediabetics	65	70	Plasma	qRT‐PCR	Down	0.91	0.64	0.756	7
Saleh et al	2020	Egypt	lncRNA GAS5	Prediabetics	65	70	Plasma	qRT‐PCR	Down	0.85	0.64	0.699	7
Saleh et al	2020	Egypt	lncRNA LY86‐AS1	Prediabetics	65	70	Plasma	qRT‐PCR	Down	0.78	0.53	0.602	7
Anbari et al	2020	Arabia	lncRNA‐GHRL‐3:2	T2DM	71	32	Blood	qRT‐PCR	Down	0.93	0.84	0.93	7
Anbari et al	2020	Arabia	lncRNA GHRL‐3:3	T2DM	71	32	Blood	qRT‐PCR	Down	0.88	0.91	0.90	7
Zhou et al	2020	China	lncRNA MALAT1	DN	27	20	PBMCs	qRT‐PCR	Up	0.80	0.68	0.79	6
Cai et al	2021	China	lncRNA SNHG5	DN	58	62	Serum	qRT‐PCR	Up	0.81	0.84	0.88	7
Rajabinejad et al	2022	Iran	lncRNA MALAT1	DN	20	20	Blood	qRT‐PCR	Up	1.00	0.71	0.92	7
Rajabinejad et al	2022	Iran	lncRNA H19	DN	20	20	Blood	qRT‐PCR	Up	0.84	0.88	0.92	7
Zhu et al	2022	China	lncRNA ANRIL	DN	66	20	Blood	qRT‐PCR	Up	0.83	0.91	0.92	7
Alfaifi et al	2021	Britain	lncRNA NKILA	DN	200	200	Serum	qRT‐PCR	Up	0.54	0.4	0.48	8
Alfaifi et al	2021	Britain	lncRNA NEAT1	DN	200	200	Serum	qRT‐PCR	Up	0.52	0.48	0.52	8
Alfaifi et al	2021	Britain	lncRNA MALAT1	DN	200	200	Serum	qRT‐PCR	Up	0.53	0.4	0.48	8
Alfaifi et al	2021	Britain	lncRNA MIAT	DN	200	200	Serum	qRT‐PCR	Up	0.55	0.5	0.53	8
Shaker et al	2018	Egypt	lncRNA HOTAIR	DR	30	30	Serum	qRT‐PCR	Up	0.76	0.8	0.78	7
Shaker et al	2018	Egypt	lncRNA MALAT1	DR	30	30	Serum	qRT‐PCR	Up	0.74	0.73	0.74	7
Liu et al	2021	China	lncRNA ENST00000505731	PDR	30	30	Blood	qRT‐PCR	Up	0.8	0.87	0.88	7
Liu et al	2021	China	lncRNA NR‐126161	PDR	30	30	Blood	qRT‐PCR	up	0.50	0.97	0.79	7
Atef et al	2022	Egypt	lncRNA HIF1A‐AS2	NPDR	15	15	PBMCs	qRT‐PCR	Up	0.89	0.92	0.94	7
Atef et al	2022	Egypt	lncRNA HIF1A‐AS2	PDR	15	15	PBMCs	qRT‐PCR	Up	0.96	0.93	0.97	7
Toraih et al	2019	Egypt	lncRNA RNCR2	DR	108	55	Plasma	qRT‐PCR	Down	0.70	0.70	0.74	6
Toraih et al	2019	Egypt	lncRNA NEAT2	DR	108	55	Plasma	qRT‐PCR	Down	0.62	0.56	0.62	6
Ji et al	2022	China	lncRNA NR2F1‐AS1	DR	158	106	Serum	qRT‐PCR	Up	0.83	0.82	0.90	7
Alfaifi et al	2021	Britain	lncRNA NKILA	DR	200	200	Serum	qRT‐PCR	Up	0.59	0.41	0.56	8
Alfaifi et al	2021	Britain	lncRNA NEAT1	DR	200	200	Serum	qRT‐PCR	Up	0.61	0.51	0.57	8
Alfaifi et al	2021	Britain	lncRNA MALAT1	DR	200	200	Serum	qRT‐PCR	Up	0.61	0.5	0.6	8
Alfaifi et al	2021	Britain	lncRNA MIAT	DR	200	200	Serum	qRT‐PCR	Up	0.68	0.58	0.66	8
Li et al	2022	China	lncRNA HIF1A‐AS1	PDR	80	100	Serum	qRT‐PCR	Up	0.66	0.79	0.77	7

Abbreviations: AUC, area under the curve; DN, diabetic nephropathy; DR, diabetic retinopathy; NOS, Newcastle‐Ottawa Scale; NPDR, nonproliferative diabetic retinopathy; PBMCs, peripheral blood mononuclear cells; PDR, proliferative diabetic retinopathy; qRT‐PCR, quantitative reverse transcription polymerase chain reaction; Sen, sensitivity; Spe, specificity; T2DM, type 2 diabetes mellitus.

### 3.2 Diagnostic value of lncRNAs for type 2 diabetes mellitus

**FIGURE 2:**
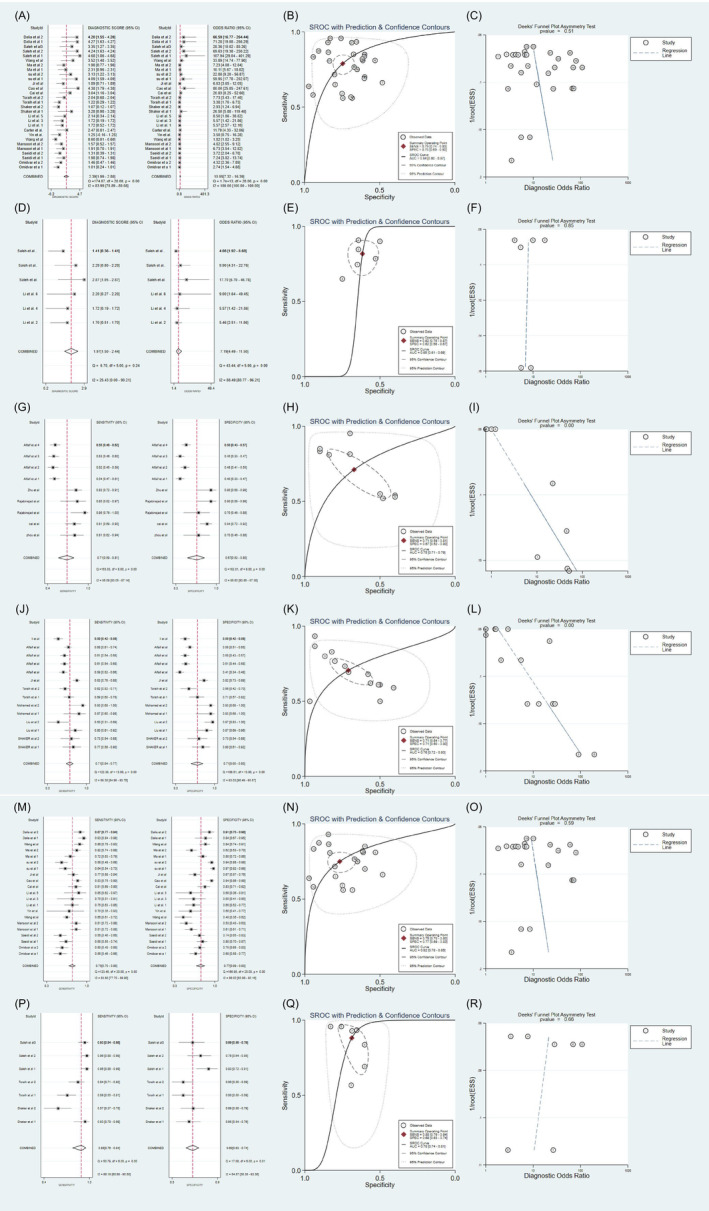
(A, D) Forest plots for sensitivity and specificity of lncRNAs for diagnosing T2DM and prediabetes; (B, E) The summary receiver operator characteristic (SROC) curve of lncRNAs for diagnosing T2DM and prediabetes; (C, F) Deeks' funnel plot asymmetry tests of lncRNAs for diagnosing T2DM and prediabetes; (G, J) Forest plots for sensitivity and specificity of lncRNAs for diagnosing diabetic nephropathy (DN) and diabetic retinopathy (DR); (H, K) The SROC curve of lncRNAs for diagnosing DN and DR; (I, L) Deeks' funnel plot asymmetry tests of lncRNAs for diagnosing DN and DR; (M, P) Forest plots for sensitivity and specificity of lncRNAs for Asia and Africa; (N, Q) The SROC curve of lncRNAs for Asia and Africa; (Q, R) Deeks' funnel plot asymmetry tests of lncRNAs for Asia and Africa. AUC, area under the curve; CI, confidence interval; ESS, effective sample sizes; lncRNA, long noncoding RNA; SENS, sensitivity; SPEC, specificity; T2DM, type 2 diabetes mellitus.

We identified 17 studies that compared patients with T2DM and healthy controls, involving a total of 4685 participants. We performed meta‐analyses on the sensitivity, specificity, positive likelihood ratio (PLR), negative likelihood ratio (NLR), and diagnostic odds ratio (DOR) of lncRNAs in diagnosing T2DM. We also compiled the summary ROC (SROC) data from these analyses. The following results were obtained by using a random‐effects model for the pooled lncRNA estimations: the AUC was 0.84 (95% CI: 0.80–0.87), and the sensitivity and specificity were 0.79 and 0.75, respectively; PLR was 3.1 (95% CI: 2.5–3.9), NLR was 0.28 (95% CI: 0.22–0.36), and DOR was 11 (95% CI: 7–16). In conclusion, lncRNA has an excellent diagnostic value for T2DM (Figure 2A,B).

### 3.3 Diagnostic value of lncRNAs for detecting prediabetes

For prediabetes, lncRNAs had the following values: AUC, 0.65 (95% CI: 0.61–0.69); sensitivity, 0.82 (95% CI: 0.75–0.87); specificity, 0.62 (95% CI: 0.56–0.67); PLR, 2.1 (95% CI: 1.8–2.5); NLR, 0.30 (95% CI: 0.21–0.42); and DOR, 7 (95% CI: 4–12). In summary, the diagnostic value of lncRNAs for prediabetes was relatively low and below the acceptable range, indicating that their diagnostic ability was unsatisfactory (Figure 2D,E).

### 3.4 Diagnostic value of lncRNAs for DN and DR

Our search results yielded 437 studies that distinguished between healthy controls and patients with DN and DR. Regarding the DN and DR studies, five eligible trials with 1913 participants and seven eligible trials with 2580 participants, respectively, were included. Summary estimates of lncRNAs were as follows: DN: AUC, 0.75; sensitivity, 71%; and specificity, 67% (Figure 2G,H), and DR: AUC, 0.7; sensitivity, 71%; and specificity, 71% (Figure 2J,K). Therefore, the diagnostic values of lncRNAs for DN and DR are acceptable.

### 3.5 Systematic review of the diagnostic value of lncRNAs for diabetic cardiomyopathy

The development and progression of diabetes lead to several problems that affect most of the major organs of the body. Diabetic cardiomyopathy (DCM) has a significant negative impact on health. We thoroughly searched all the literature on the value of lncRNAs for detection of DCM and found 25 studies, 23 of which were reviews or animal studies, and one study did not report diagnostic results.^40^ Ultimately, only one study met the inclusion criteria; thus, we were unable to perform a meta‐analysis. Regarding the diagnosis of DCM, the AUC values for terminal differentiation‐induced ncRNA expression in myocardial biopsy and serum samples were 0.95 (95% CI: 0.89–1.0) and 0.93 (95% CI: 0.85–1.0), respectively. Therefore, downregulation of terminal differentiation‐induced ncRNA expression may be a potential diagnostic biomarker for DCM.^41^

### 3.6 Subgroup analysis based on ethnic derivation

The validity of lncRNAs as diagnostic tools may vary according to dietary differences across ethnicities. We also performed a subgroup analysis of the lncRNA expression levels in various ethnic groups. The random‐effects model yielded an AUC of 0.82 (95% CI: 0.78–0.85), sensitivity of 0.75 (95% CI: 0.70–0.80), and specificity of 0.77 (95% CI: 0.69–0.83) among Asian participants (Figure 2M,N). For participants from Africa, these values were 0.78 (95% CI: 0.74–0.81), 0.88 (95% CI: 0.76–0.94), and 0.69 (95% CI: 0.63–0.74), respectively (Figure 2P,Q).

### 3.7 Subgroup analysis based on lncRNA levels

Using the data on the differential expression of lncRNA, all included studies were divided into two groups based on the abnormal expression of lncRNAs, including nine downregulated and eight upregulated lncRNAs. As for the downregulated lncRNAs, the AUC of the SROC curve was 0.81 (95% CI: 0.77–0.84), with a sensitivity of 0.81 (95% CI: 0.72–0.87) and a specificity of 0.71 (95% CI: 0.66–0.77; Supplementary S1A,B). Regarding the upregulated lncRNAs, the AUC of the SROC curve was 0.83 (95% CI: 0.80–0.86), with a sensitivity of 0.77 (95% CI: 0.71–0.82) and a specificity of 0.78 (95% CI: 0.68–0.86; Supplementary S1D,E).

### 3.8 Subgroup analysis based on the sample type

To examine the sources of heterogeneity in the different diagnostic values of lncRNAs for the detection of T2DM, we performed a subgroup analysis based on the source of specimens tested for lncRNA expression levels.

According to the sample type, all included studies were classified into four groups: PBMCs, plasma, serum, and blood. For the PBMC group, the results of the random‐effects model were as follows: an AUC of 0.76 (95% CI: 0.72–0.79), sensitivity of 0.70 (95% CI: 0.62–0.77), and specificity of 0.70 (95% CI: 0.63–0.76; Supplementary S2A,B). For the blood group, the results showed an AUC of 0.87 (95% CI: 0.83–0.89), sensitivity of 0.82 (95% CI: 0.76–0.87), and specificity of 0.77 (95% CI: 0.69–0.83; Supplementary S2D,E). For the plasma group, the results reported an AUC of 0.81 (95% CI: 0.77–0.95), sensitivity of 0.89 (95% CI: 0.77–0.95), and specificity of 0.68 (95% CI: 0.60–0.76; Supplementary S2G,H). For the serum group, the results showed an AUC of 0.89 (95% CI: 0.86–0.91), sensitivity of 0.77 (95% CI: 0.66–0.85), and specificity of 0.87 (95% CI: 0.77–0.94); (Supplementary S2J,K).

### 3.9 Risk of bias and sensitivity analysis

Funnel plots showed no evidence of publication bias in our meta‐analysis. Moreover, we performed a sensitivity analysis to assess the reliability of our meta‐analysis results by excluding one study at a time, and no outliers were found. Investigating the source of heterogeneity is challenging, and this may be the cause of all detected heterogeneity.

## 4 DISCUSSION

**FIGURE 3:**
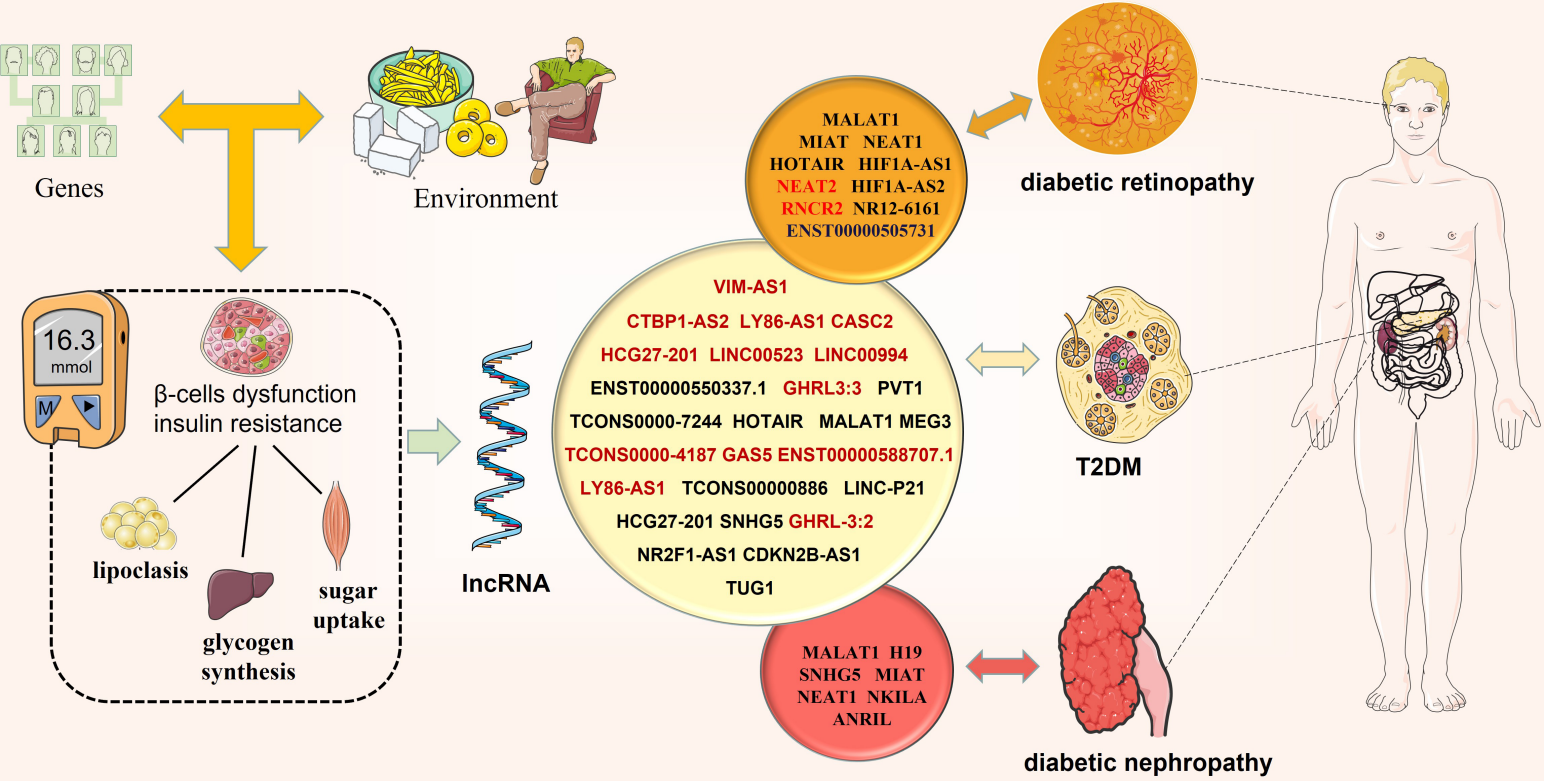
Genes, environment, and other factors cause β‐cells dysfunction and insulin resistance, leading to abnormal increase of blood glucose level, and eventually cause diabetic retinopathy, diabetic nephropathy, and other microvascular lesions. The legend demonstrates the summarized lncRNAs with a diagnostic predictive value for type 2 diabetes mellitus (T2DM), diabetic retinopathy and diabetic nephropathy, where the typical red lncRNAs are the downregulated lncRNAs. lncRNA, long noncoding RNA. [Correction added on 2 February 2024, after first online publication: we have interchanged the images of figures 3 and 4.]

We conducted a systematic review and meta‐analysis of case–control and cohort studies on the association between lncRNA expression and diagnostic outcomes in patients with T2DM or its associated complications (see the schema in Figure 3).

Our study revealed a significant correlation between the expression of abnormal lncRNAs and the presence of T2DM, which is consistent with the findings of previous studies.^42^ In our meta‐analysis, we included 10 studies more than the seven included in a previous study.^42^ We discovered that lncRNAs are highly accurate in diagnosing T2DM or prediabetes. In addition, we analyzed five and seven lncRNA studies on DN and DR, respectively, and our findings revealed that lncRNAs have some diagnostic power for the detection of DN and DR. We performed a regression analysis to investigate the causes of heterogeneity and did not identify any outliers. Using subgroup analysis, we found that lncRNAs had a unique diagnostic predictive value for T2DM across sample sources and ethnic differences, with high diagnostic value in serum samples and in samples from Asian populations.

Inflammation and dysregulated metabolism affect multiple cells and organs, such as beta cells, and liver, skeletal muscle, kidney, brain, and adipose tissue, gradually resulting in T2DM.^43^ Cao et al^31^ suggested that serum expression of LINC‐P21 was elevated in patients with T2DM, related to its targeting of miR‐766‐3p to upregulate NR3C2, resulting in insulin secretion and proliferation in pancreatic beta cells. Elevated serum LINC‐P21 and decreased serum miR‐766‐3p levels are candidate diagnostic biomarkers in patients with T2DM. Studies investigating the upregulation or inhibition of lncRNA in T2DM revealed an association with functional impairment of INS‐1 cells or increased hepatic glycogen synthesis.^44^ In general, these studies suggest that lncRNAs have the potential as direct targets for therapeutic interventions in diabetes. Thus, lncRNAs have emerged as important regulators of glucose and lipid metabolism.

Moreover, the role of lncRNAs in diabetes‐related complications should not be overlooked. One of the most serious microvascular complications of DM is DN due to mesangial cell proliferation and changes in the renal microenvironment.^45^ In an animal study, the lncRNA SOX2OT was significantly downregulated in the mesangial cells by different pathways, whereas overexpression thereof significantly inhibited fibrosis of the mesangial cells.^46^ In a study by Cai et al, SNHG5 expression was substantially elevated in DN, confirming its potential as a novel biomarker for the diagnosis of DN that may interact with miR‐26a‐5p. DN is characterized by an accumulation of extracellular matrix, hypertrophy, and fibrosis in the glomeruli and renal tubular cells. Additionally, growing evidence suggests that these DN features are closely linked to ncRNA regulation.^47^

Ogurtsova et al^48^ reported that lncRNAs have unique expression profiles and play an important role in the development of DR. One study found that lncRNA H19 blocked endothelial‐mesenchymal transition (EndMT) in DR.^49^ It inhibited transforming growth factor‐1 and its signaling pathway by blocking the MAPK‐ERK1/2 signaling pathway that controlled EndMT when glucose levels are elevated. A similar mechanistic study of lncRNAs in DR was reported, in which overexpression of the lncRNA SNHG7 inhibited EndMT and tube formation.^50^

Our study demonstrated the applicability of lncRNAs as diagnostic biomarkers not only for T2DM but also for its complications, such as DN and DR.

We conducted a meta‐regression and subgroup analysis based on ethnic origin, sample origin, and lncRNA levels. To improve the search for biomarkers for early diagnosis of type 2 diabetes, future studies should include subgroup analyses based on specific types of lncRNA, sex, and age. Fortunately, obtaining patient blood samples and determining the levels of lncRNA expression make this investigation easy and practical. Furthermore, this diagnostic analysis showed no asymmetry in the Deeks' funnel plot, indicating no publication bias. The high incidence and prevalence of T2DM poses challenges to the effective treatment of patients. Therefore, early detection of diabetes and prevention of its complications are particularly important for high‐risk groups.

**FIGURE 4:**
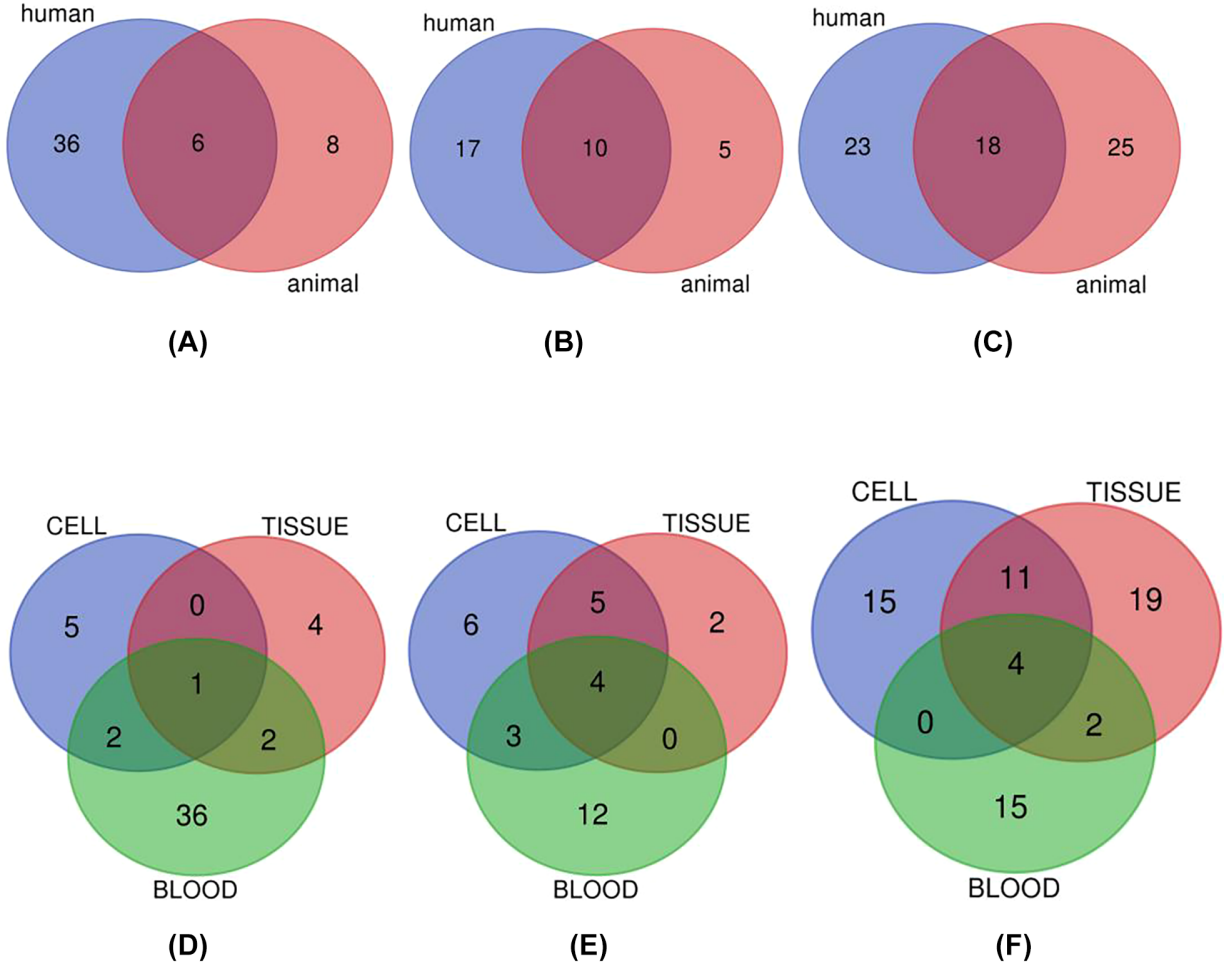
Venn diagram of the overlapping number of lncRNAs differentially expressed by type 2 diabetes mellitus (T2DM) (A), diabetic retinopathy (DR) (B), diabetic nephropathy (DN) (C) among studies conducted in humans and animals. Venn diagram of the overlapping number of lncRNAs differentially expressed by T2DM (D), DR (E), DN (F) among studies conducted in cell, tissue, and blood subjects. lncRNA, long noncoding RNA.

A large number of lncRNAs have been tested in animal models but not in human tissues. This may be attributed to the lower expense of animal experiments compared with that of clinical studies. Owing to the limited resources available for clinical studies, we aimed to validate lncRNA biomarkers that are prevalent in animals and humans by using Venn diagram statistics. As shown in Figure 4, different biomarkers have been detected in humans and animals or in blood, tissues, and cells. Our Venn diagrams show that MALAT1 and TUG1 are examples of the lncRNAs validated in animals and humans, whereas MALAT1 and NEAT1 are examples of those validated in diabetic cells, tissues, and blood. These findings may serve as a solid starting point for future studies. Table 2 provides specific information on the characteristics of the overlapping lncRNAs. To the best of our knowledge, this is the first meta‐analysis to explore the diagnostic value of lncRNAs in T2DM, DN, and DR.

**TABLE 2:** Venn diagram detail table for the lncRNAs overlap numbers.

Item	Names	Total	Elements
T2DM	Human ∩ animal	6	EPB41L4A‐AS1 MALAT1 TUG1 DRAIR MIAT Kcnq1ot1
DR	Human ∩ animal	10	NEAT1 MALAT1 HOTAIR H19 XIST ZFAS1 MEG3 ANRIL TUG1 AQP4‐AS1
DN	Human ∩ animal	18	CYP4B1‐PS1–001 SOX2OT HOXB3OS NEAT1 GAS5 XIST Lnc‐ISG20 CASC2 PVT1 OIP5–AS1 H19 MEG3 NONHSAG053901 TUG1 CES1P1 MIAT ANRIL MALAT1
T2DM	Cell ∩ tissue ∩ blood	1	MALAT1
DR	Cell ∩ tissue ∩ blood	4	HOTAIR ZFAS1 MEG3 ANRIL
DN	Cell ∩ tissue ∩ blood	4	NEAT1 CASC2 MALAT1 ANRIL

Abbreviations: DN, diabetic nephropathy; DR, diabetic retinopathy; lncRNA, long noncoding RNA; T2DM, type 2 diabetes mellitus.

## 5 LIMITATIONS AND FUTURE RECOMMENDATIONS

Our meta‐analysis has some limitations. First, some heterogeneity was detected, and subgroup analysis based on the lncRNA type could not fully explore the main sources of heterogeneity owing to the limited number of studies. Second, the sample size of studies on the diagnostic value of lncRNAs in DN and DR was relatively small, which may have led to insufficient statistical power. Third, the sample size of the lncRNA studies on other diabetic complications was insufficient for a meta‐analysis, allowing only systematic assessment; thus, their associated diagnostic value requires further study. Fourth, although a large number of lncRNAs have been tested in humans, the experimental results did not provide specific AUCs, sensitivities, or specificities. Despite our attempts to obtain these data by email, the results were not available; therefore, we were unable to obtain additional data for the meta‐analysis.

Recent studies have suggested that lncRNAs, circular RNAs, and mRNAs compete with each other through miRNA response elements to modulate the progression of T2DM and may function as miRNA sponges.^51^ Thus, future research may focus on constructing a competing endogenous RNA network to further understand the biological effects of lncRNAs in patients with T2DM and its complications. Furthermore, lncRNAs may compete for shared miRNAs to regulate other RNA transcripts, thereby influencing the pathogenesis of T2DM and its complications. Finally, with a large number of lncRNAs studies in the diagnosis of diabetes, we may focus on the diagnostic value of a specific LncRNA for diabetes and related complications.

## 6 CONCLUSION

Our findings suggest that lncRNAs are promising biomarkers and therapeutic targets for T2DM, DN, and DR. They may be extremely beneficial in the early detection and diagnosis of these conditions. The biological functions of these markers require further investigation to help determine the molecular mechanisms underlying the pathogenesis of T2DM.

## AUTHORS CONTRIBUTIONS

Xuee Su, Huibin Huang, Yinqiong Huang, and Shu Lin were involved in the conception of the article. Xuee Su and Jinqing Lai independently examined studies that might be eligible for inclusion, and any disagreements were resolved by a third review author (Yinqiong Huang), if necessary. Xuee Su and Jinqing Lai obtained datasets for research based on a standardized form. After the consensus was reached, the data were entered into STATA statistical software. and any uncertainties were discussed and highlighted (Yinqiong Huang and Shu Lin). Xuee Su and Huibin Huang drafted the manuscript. Yinqiong Huang and Shu Lin contribute to project supervision, and study design. All authors have read and approved the final manuscript. Xuee Su and Huibin Huang contributed equally to this work.

## FUNDING INFORMATION

This work was supported by the Science and Technology Bureau of Quanzhou (grant number 2020CT003), the Nursery Fund Project of the Second Affiliated Hospital of Fujian Medical University (2021MP25), Key Young Talents Health Training Project of Fujian Province (2020GGA057), Natural Science Foundation of Fujian Province (2020J01221), and National Natural Science Foundation of China (82200871). The funders had no role in study design, data collection, data analysis, data interpretation, or writing of the report.

## DISCLOSURE

The authors declare no competing fnancial interests.

## Supporting information


**Data S1.** Supporting Information.

## Data Availability

This article (and its supplementary information files) contains all data generated or analyzed during this study.
