# Supplementary material for: Long noncoding RNAs as potential diagnostic biomarkers for diabetes mellitus and complications: A systematic review and meta‐analysis
Source: J Diabetes. 2023 Dec 23;16(2):e13510. doi: 10.1111/1753-0407.13510 (PMC10847882; doi:10.1111/1753-0407.13510)
Supplement: Supplementary file 1 — Data S1. Supporting Information. [file JDB-16-e13510-s001.docx]

Suppl. 1 A, D Forest plots for sensitivity and specificity of downregulated and upregulate lncRNAs for diagnosing T2DM; B, E The summary receiver operator characteristic (SROC) curve of downregulated and upregulated lncRNAs for diagnosing T2DM; C, F Deeks’funnel plot asymmetry tests of downregulated and upregulated lncRNAs for diagnosing T2DM
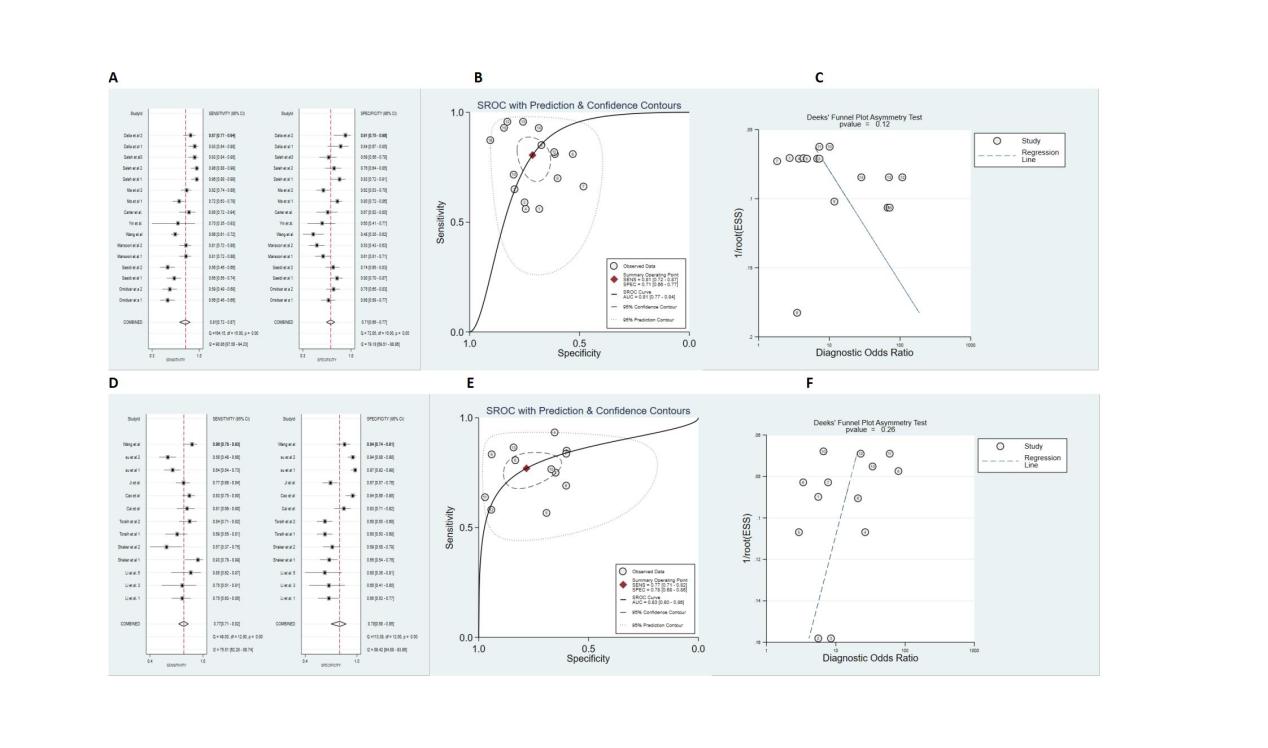


Suppl. 2 A, D, G, J Forest plots for sensitivity and specificity of lncRNAs for PBMCs, blood, plasma, serum specimen diagnosing T2DM; B, E, H, K The summary receiver operator characteristic (SROC) curve of lncRNAs for PBMCs, blood, plasma, serum specimen diagnosing T2DM diagnosing T2DM; C, F, I, L Deeks’ funnel plot asymmetry tests of lncRNAs for PBMCs, blood, plasma, serum specimen diagnosing T2DM diagnosing T2D
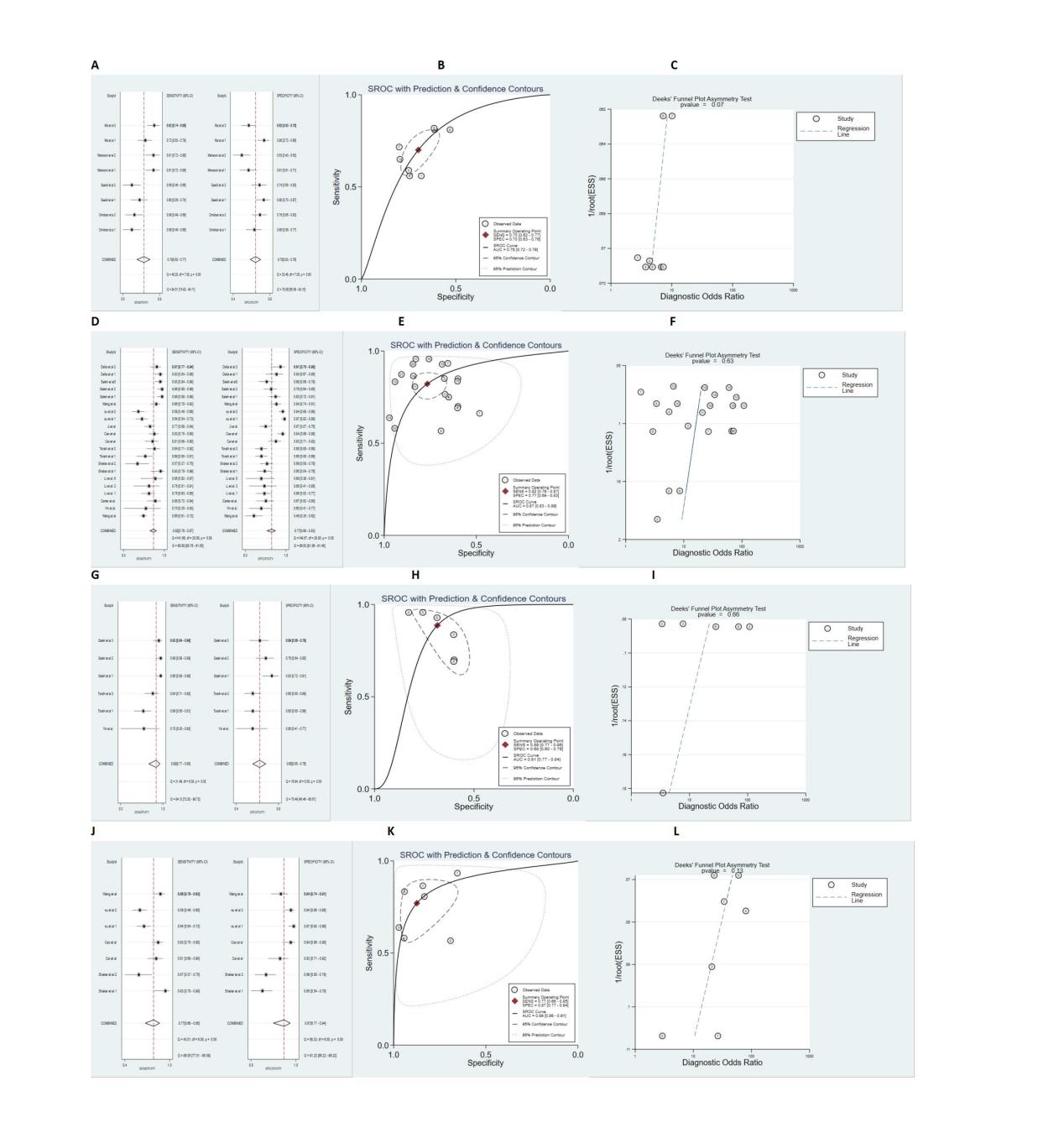


Suppl. 3 Search strategies for databases including PubMed, Embase and Web of Science

1. **PubMed**

| **No.** | **Query** | **Results** |
| --- | --- | --- |
| #5 | #1 AND #4 | 661 |
| #4 | #2 OR #3 | 306695 |
| #3 | (((((Diabetes Complications[MeSH Terms]) OR (Diabetes Complication[Title/Abstract])) OR (Diabetes-Related Complications[Title/Abstract])) OR (Complications of Diabetes Mellitus[Title/Abstract])) OR (Diabetes Mellitus Complication[Title/Abstract])) OR (Diabetes Mellitus Complications[Title/Abstract]) | 117787 |
| #2 | ((((((((((((((Diabetes Mellitus, Type 2[MeSH Terms]) OR (Diabetes Mellitus, Noninsulin-Dependent[Title/Abstract])) OR (Diabetes Mellitus, Ketosis-Resistant[Title/Abstract])) OR (Ketosis-Resistant Diabetes Mellitus[Title/Abstract])) OR (Diabetes Mellitus, Non Insulin Dependent[Title/Abstract])) OR (Non-Insulin-Dependent Diabetes Mellitus [Title/Abstract])) OR (Diabetes Mellitus, Stable[Title/Abstract])) OR (Stable Diabetes Mellitus[Title/Abstract])) OR (Diabetes Mellitus, Type II[Title/Abstract])) OR (Diabetes Mellitus, Maturity-Onset[Title/Abstract])) OR (Diabetes Mellitus, Slow Onset[Title/Abstract])) OR (Type 2 Diabetes Mellitus[Title/Abstract])) OR (Type 2 Diabetes[Title/Abstract])) OR (Diabetes, Type 2[Title/Abstract])) OR (Diabetes Mellitus, Adult-Onset[Title/Abstract]) | 218167 |
| #1 | (((((((((((((((((((((((((RNA, Long Noncoding[MeSH Terms]) OR (Noncoding RNA[Title/Abstract])) OR (Long lncRNA[Title/Abstract])) OR (Long ncRNA[Title/Abstract])) OR (ncRNA, Long[Title/Abstract])) OR (RNA, Long Non-Translated[Title/Abstract])) OR (Long Non-Translated RNA[Title/Abstract])) OR (Non-Translated RNA, Long[Title/Abstract])) OR (RNA, Long Non Translated[Title/Abstract])) OR (Long Non-Coding RNA[Title/Abstract])) OR (Non-Coding RNA, Long[Title/Abstract])) OR (RNA, Long Non-Coding[Title/Abstract])) OR (Long Non-Protein-Coding RNA[Title/Abstract])) OR (Long Non Protein Coding RNA[Title/Abstract])) OR (Non-Protein-Coding RNA, Long[Title/Abstract])) OR (RNA, Long Non-Protein-Coding[Title/Abstract])) OR (Long Noncoding RNA[Title/Abstract])) OR (RNA, Long Untranslated[Title/Abstract])) OR (Long Untranslated RNA[Title/Abstract])) OR (Untranslated RNA, Long[Title/Abstract])) OR (Long ncRNAs[Title/Abstract])) OR (ncRNAs, Long[Title/Abstract])) OR (Long Intergenic Non-Protein Coding RNA[Title/Abstract])) OR (LincRNAs[Title/Abstract])) OR (LINC RNA[Title/Abstract])) OR (LincRNA[Title/Abstract]) | 42801 |

1. **Embase**

| **No.** | **Query** | **Results** |
| --- | --- | --- |
| #9 | #15 AND #16 | 1271 |
| #8 | #3 OR #4 OR #5 OR #6 | 505571 |
| #7 | #1 OR #2 OR | 55343 |
| #6 | 'diabetes complications':ti,ab,kw OR 'diabetes-related complications':ti,ab,kw OR 'complications of diabetes mellitus':ti,ab,kw OR 'diabetes mellitus complication':ti,ab,kw | 11490 |
| #5 | 'diabetes complications' OR 'diabetes complications'/exp | 202845 |
| #4 | 'diabetes mellitus, type 2':ti,ab,kw OR 'diabetes mellitus, noninsulin-dependent':ti,ab,kw OR 'diabetes mellitus, ketosis-resistant':ti,ab,kw OR 'diabetes mellitus, non insulin dependent':ti,ab,kw OR 'non-insulin-dependent diabetes mellitus':ti,ab,kw OR 'diabetes mellitus, type ii':ti,ab,kw OR 'diabetes, type 2':ti,ab,kw | 21652 |
| #3 | 'diabetes mellitus, type 2' OR 'diabetes mellitus, type 2'/exp | 344548 |
| #2 | ''rna, long noncoding':ti,ab,kw OR 'noncoding rna':ti,ab,kw OR 'long lncrna':ti,ab,kw OR 'long ncrna':ti,ab,kw OR 'rna, long non-translated':ti,ab,kw OR 'non-translated rna, long':ti,ab,kw OR lincrna:ti,ab,kw | 18087 |
| #1 | 'rna, long noncoding'/exp OR 'rna, long noncoding | 48,357 |

1. **Web of Science**

| **No.** | **Query** | **Results** |
| --- | --- | --- |
| #9 | #1 AND #4 | 455 |
| #4 | '#2 OR #3 | 181141 |
| #3 | (((((((((((((((TS=(diabetes complications)) OR TI=(Diabetes Complication)) OR TI=(Diabetes-Related Complications)) OR TI=(Complications of Diabetes Mellitus)) OR TI=(Diabetes Mellitus Complication)) OR TI=(Diabetes Mellitus Complications)) OR AB=(Diabetes Complication)) OR AB=(Diabetes-Related Complications)) OR AB=(Complications of Diabetes Mellitus)) OR AB=(Diabetes Mellitus Complication)) OR AB=(Diabetes Mellitus Complications)) OR KP=(Diabetes Complication)) OR KP=(Diabetes-Related Complications)) OR KP=(Diabetes Mellitus Complications)) OR KP=(Diabetes Mellitus Complication)) OR KP=(Diabetes Mellitus Complications) | 80949 |
| #2 | (((((((((((((((TS=(Diabetes Mellitus, Type 2)) OR TI=(Diabetes Mellitus, Noninsulin-Dependent)) OR TI=(Diabetes Mellitus, Ketosis-Resistant)) OR TI=(Ketosis-Resistant Diabetes Mellitus)) OR TI=(Diabetes Mellitus, Non Insulin Dependent)) OR TI=(Type 2 Diabetes Mellitus)) OR AB=(Diabetes Mellitus, Noninsulin-Dependent)) OR AB=(Diabetes Mellitus, Ketosis-Resistant)) OR AB=(Ketosis-Resistant Diabetes Mellitus)) OR AB=(Diabetes Mellitus, Non Insulin Dependent)) OR AB=(Type 2 Diabetes Mellitus)) OR KP=(Diabetes Mellitus, Noninsulin-Dependent)) OR KP=(Diabetes Mellitus, Ketosis-Resistant)) OR KP=(Ketosis-Resistant Diabetes Mellitus)) OR KP=(Diabetes Mellitus, Non Insulin Dependent)) OR KP=(Type 2 Diabetes Mellitus) | 120532 |
| #1 | (((((((((((((((TS=(RNA, Long Noncoding)) OR TI=(Noncoding RNA))) OR TI=(Long lncRNA)) OR TI=(Long ncRNA)) OR TI=(ncRNA, Long)) OR TI=(RNA, Long Non-Translated)) OR AB=(Noncoding RNA)) OR AB=(Long lncRNA)) OR AB=(Long ncRNA)) OR AB=(ncRNA, Long)) OR AB=(RNA, Long Non-Translated)) OR KP=(Noncoding RNA)) OR KP=(Long lncRNA)) OR KP=(Long ncRNA)) OR KP=(RNA, Long Non-Translated) | 53864 |

Suppl. 4 Results of quality assessment using the Newcastle-Ottawa Scale

| Study | Selection | | | | Comparability | Outcome | | | Scores |
| --- | --- | --- | --- | --- | --- | --- | --- | --- | --- |
|  | Representativeness of the exposed cohort | Selection of the non-exposed cohort | Ascertainment of exposure | Demonstration that outcome of interest was not present at start of study | Comparability of cohorts on the basis of the design or analysis | Assessment of outcome | Was follow-up long enough for outcomes to occur | Adequacy of follow-up of cohorts |  |
| Omidvar et al,2018 | 1 | 1 | 1 | 1 | 2 | 0 | 1 | 0 | 7 |
| Saeidi et al,2018 | 0 | 1 | 1 | 1 | 1 | 1 | 1 | 1 | 7 |
| Mansoori et al,2018 | 1 | 1 | 1 | 1 | 1 | 0 | 1 | 1 | 7 |
| Wang et aL,2018 | 1 | 1 | 1 | 1 | 1 | 0 | 1 | 0 | 6 |
| Yin et al,2017 | 0 | 1 | 1 | 1 | 1 | 1 | 1 | 1 | 7 |
| Carter et al,2015 | 0 | 1 | 1 | 1 | 2 | 0 | 1 | 1 | 7 |
| Li et al,2017 | 0 | 1 | 1 | 1 | 1 | 1 | 1 | 0 | 6 |
| Shaker et al,2018 | 1 | 1 | 1 | 1 | 2 | 0 | 1 | 0 | 7 |
| Toraih et al,2019 | 1 | 1 | 1 | 1 | 1 | 0 | 1 | 0 | 6 |
| Cai et a,2021 | 0 | 1 | 1 | 1 | 1 | 1 | 1 | 0 | 7 |
| Cao et al,2020 | 0 | 1 | 1 | 1 | 1 | 0 | 1 | 1 | 6 |
| Ji et al,2022 | 1 | 1 | 1 | 1 | 1 | 1 | 1 | 0 | 7 |
| Su et al, 2022 | 1 | 1 | 1 | 1 | 1 | 0 | 1 | 1 | 7 |
| Ma et al,2021 | 1 | 1 | 1 | 1 | 2 | 0 | 1 | 0 | 7 |
| Wang et al,2021 | 0 | 1 | 1 | 1 | 2 | 1 | 1 | 1 | 8 |
| Saleh et al,2020 | 1 | 1 | 1 | 1 | 1 | 0 | 1 | 1 | 7 |
| Anbari et al,2020 | 1 | 1 | 1 | 1 | 1 | 1 | 1 | 0 | 7 |
| Zhou et al,2020 | 0 | 1 | 1 | 1 | 1 | 1 | 1 | 0 | 6 |
| Rajabinejad et al,2022 | 1 | 1 | 1 | 1 | 1 | 1 | 1 | 0 | 7 |
| Zhu et al,2022 | 1 | 1 | 1 | 1 | 2 | 0 | 1 | 0 | 7 |
| Alfaifi et a,2021 | 1 | 1 | 1 | 1 | 1 | 1 | 1 | 1 | 8 |
| Liu et al,2021 | 0 | 1 | 1 | 1 | 1 | 1 | 1 | 1 | 7 |
| Atef et al,2022 | 1 | 1 | 1 | 1 | 1 | 1 | 1 | 0 | 7 |
| Li et al,2022 | 1 | 1 | 1 | 1 | 1 | 0 | 1 | 1 | 7 |
